# Tribological Properties and Lubrication Mechanism of Nickel Nanoparticles as an Additive in Lithium Grease

**DOI:** 10.3390/nano12132287

**Published:** 2022-07-03

**Authors:** Jiabei Wang, Hong Zhang, Wenjing Hu, Jiusheng Li

**Affiliations:** 1 Laboratory for Advanced Lubricating Materials, Shanghai Advanced Research Institute, Chinese Academy of Sciences, Shanghai 201210, China; wangjiabei2019@sari.ac.cn (J.W.); xyyyswlp@163.com (H.Z.); 2 University of Chinese Academy of Sciences, Beijing 100049, China

**Keywords:** nano-lubrication, lithium grease, additives, nanometer Ni, tribological performance

## Abstract

Nanomaterials exhibit intriguing tribological performance and have received particular attention in the lubrication field. However, little research has been found that surveyed the application of nanometer Ni in lithium grease. In this study, nanometer Ni with an average size of 100 nm was synthesized by the direct reduction method and dispersed in lithium grease. The feasibility of nanometer Ni as a grease additive in different lubrication scenarios was evaluated by a four-ball friction tester and a TE77 reciprocating friction tester. The lubrication mechanism was analyzed based on the evaluated physical properties of lithium grease and the characterization of the wear surface. The tribology test results showed the tribological properties of lithium grease were enhanced after introducing nanometer Ni. When the dosage was 0.2 wt%, the friction-reducing and anti-wear properties of point-to-point contact increased by 34.8% and 35.2%, respectively, while those of the point-to-flat contact increased by 28.8% and 38.7%, respectively. Our work not only provides theoretical guidance and practical reference for the utilization of nanometer Ni in grease, but also explains several possible lubrication mechanisms of nanomaterials in grease.

## 1. Introduction

Devising energy-efficient machinery with low energy consumption, low emission, and high power is highly demanded for the sustainable development of the economy [1]. Friction and wear play critical roles in the issue of energy loss and parts damage in moving machinery systems. Lubricants, as the primary means, form viscous lubrication films and rigid edge membranes at the contact interfaces, preventing direct asperity interactions and thereby reducing the possibility of surface adhesion, abrasion and fatigue wear [2,3,4]. The two most popular lubricants in the industry field are lubricating oil and grease. Although lubricating oils possess excellent fluidity which can induce a dynamic pressure effect and fully lubricate fine structures [5,6], further applications are hindered by the non-uniformity of oil films. By contrast, grease is more applicable in certain cases owing to its semisolid characteristic, which helps to protect equipment from pollutants and reduce noise. Due to its excellent tolerance of water and anti-rust property, lithium grease is one of the most commonly used greases in the industry [7,8,9]. Lithium grease is composed of colloidal suspensions formed by dispersing thickeners and additives in mineral or synthetic oils. The thickener fiber has a typical network structure, which exerts a certain influence on the friction-reducing and anti-wear properties. Previous research has established the lubrication mechanism of the thickener. In addition to directly participating in the formation of lubricating film, it affects the lubrication process by controlling the base oil, which can be represented by the capacity of oil separation. Meanwhile, base oil and additives are critical to the tribology properties of grease [10]. 

With the development of nanoscience and technology [11], metallic nanoparticles with unique physical and chemical properties have become effective additives to lubricant grease [12,13]. The metallic nano additives provide normal greases with some obvious advantages such as the abilities of lubricant retention and wear resistance. Compared with traditional organic long-chain additives, metal nanoparticles are easier to mix and bring fewer chemical products into the friction process. Meng et al. [14] demonstrated that a mixture of nanometer Cu and lubricating oil can form a protective film with nanometer Cu on the friction surface, which increased the operating life of mechanical parts. Yu et al. [15] revealed that nanometer Cu, as a lubricant additive, has a significant effect on the tribological performance of lubricating systems. Wang et al. [16] demonstrated that utilizing CaF_2_ nanocrystals as a lubricating additive in grease prompts excellent friction-reducing and anti-wear properties. Due to its corrosion resistance, oxidation resistance, ferromagnetism, and high recycling efficiency [17], nanometer Ni has been widely studied and applied in various scenarios. The research on nanometer Ni will help develop more high-end lubricating additives based on Ni in theoretical research. However, since metallic nano additives cannot achieve stable and uniform dispersion in liquid lubricants, nanometer Ni is mainly used to improve the tribological properties of steel coatings by forming composites with other organic compounds. Sundararajan et al. [18] determined that using Ni-P in anodic aluminum oxide effectively solved the scratch failure problem of electronic packaging surfaces. Wang et al. [19] reported that a double-layer Ni-P-Cr composite coating was beneficial in improving the mechanical and anticorrosion properties. Zhu et al. [20] proved the excellent lubricating performance of Cu-15 wt%Ni-8 wt%Sn bronze alloy as an additive in the bearing field. The research to date has tended to focus on modified nanometer Ni rather than pure nanometer Ni.

A considerable amount of literature demonstrates the great promise of nanometer Ni in lubrication. Considering the dispersion stability of nanometer Ni in lubricating oil, and its toxicity [21], grease was chosen as a dispersion system to study the tribological properties of nanometer Ni. Nanometer Ni can achieve a stable dispersion in semi-solid grease, and its replacement frequency in practical use is also lower than that of liquid lubricant. This paper attempts to determine the friction-reducing and anti-wear properties of lithium grease with nanometer Ni as an additive. The nanometer Ni with loose agglomeration was synthesized by the direct reduction method. The Ni-doped lithium grease with different concentrations was prepared by uniformly mixing nanometer Ni and pure grease, and the tribology properties and various physical properties were measured. The composition and structure of worn surfaces were investigated under various lubricating conditions. We also aim to explore the optimal content of nanometer Ni in doped lithium grease with the best friction-reducing and anti-wear properties, and further address the lubricating mechanism of nanomaterials on this basis.

## 2. Materials and Methods

### 2.1. Materials

In this study, lithium grease (Hupai Petroleum Co., Ltd., Ningbo, China) was selected as the base grease. Poly alpha olefins (PAO4) were purchased from Formosa Petrochemical Co., Ltd., China. Nickel (II) formate dihydrate (Ni(HCOO)_2_·2H_2_O) was purchased from Shanghai Aladdin Co., Ltd. (Shanghai, China). The analytical reagents, titanium butoxide, alcohol, and petroleum ether (PE) were purchased from Sinopharm Chemical Reagent Co., Ltd., Shanghai, China. To ensure easy commercialization, all of the raw materials are available in the commercial market without further purification.

### 2.2. Preparation of Nanometer Ni and Ni-Doped Lithium Grease

#### 2.2.1. Synthesis of Nanometer Ni

**Figure 1:**
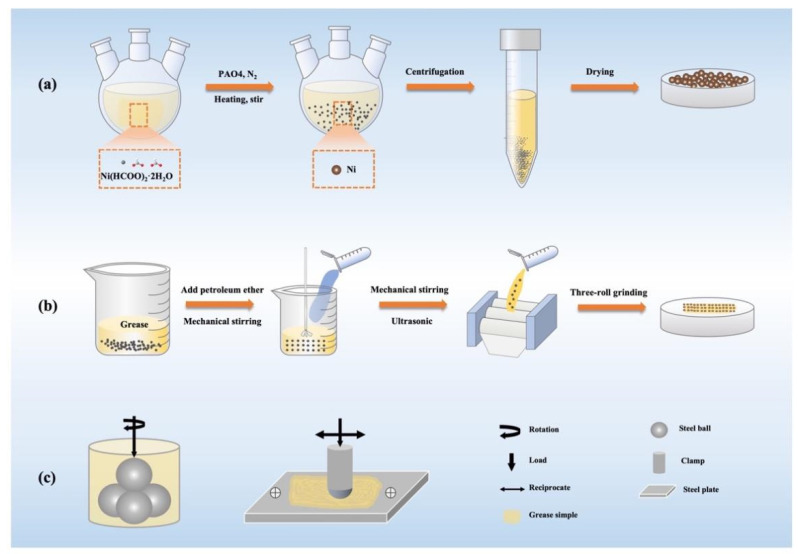
The schematic diagram of the synthesis process of (**a**) nanometer Ni and (**b**) Ni-doped lithium grease; (**c**) the schematic diagram of tribological test arrangement.

Ni(HCOO)_2_·2H_2_O (10 g, 54.1 mmol) and the oil of PAO4 (40 mL) were added to the three-port round bottom flask and raised the temperature to 230 °C directly. The whole reaction was completed in N_2_ to ensure an anhydrous and anaerobic environment. After the reaction was cooled to room temperature, the solution was separated from the oil and nanoparticles through centrifugal under 6000 rpm/min for 3 min. The obtained black product was washed with PE and alcohol and weighed after drying at 60 °C for 5 h. Figure 1a presents the flow chart for the synthesis of nanometer Ni.

#### 2.2.2. Preparation of Lithium Grease with Additive

**Figure 2:**
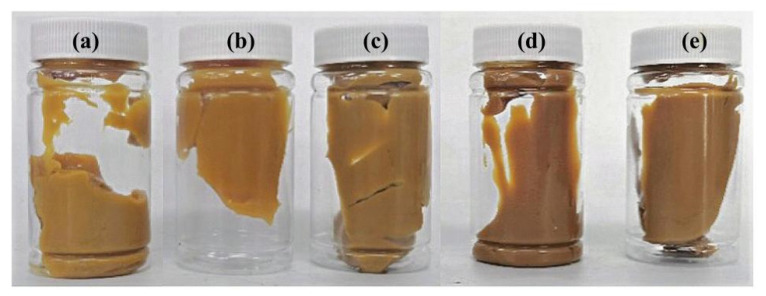
Lithium grease with nanometer Ni in various concentrations (wt%): (**a**) 0, (**b**) 0.05, (**c**) 0.1, (**d**) 0.2, (**e**) 0.3.

The preparation process of Ni-doped lithium grease is also illustrated in Figure 1b. First of all, the instruments were cleaned with PE and alcohol, and dried to ensure that there were no impurities. Secondly, different amounts of nano-Ni were added to pure lithium grease to attain the doped lithium grease with various concentrates (0, 0.05 wt%, 0.1 wt%, 0.2 wt%, 0.3 wt%). Each grease sample was dissolved with 15 mL of PE. After mechanical stirring of the mixture at 50 ℃ for 60 min, the PE was completely evaporated. Subsequently, the uniformly doped lithium grease was obtained after ultrasonic vibration for 30 min. Finally, the grease was milled three times by a three-roll milling machine. The final products are shown in Figure 2.

### 2.3. Physical and Tribological Characterization of Grease

#### 2.3.1. Physical Characterization Tests of Grease

The physical characterizations of the pure grease and 0.2 wt% Ni-doped grease were investigated by national standards. The properties measured were cone penetration, drop point, and oil separation rate. Table 1 tabulates the test conditions. All tests were conducted three times to ensure reliability, and the average data are described.

#### 2.3.2. Tribological Tests of Grease

The tribological behavior of grease was tested on a four-ball friction tester under point-to-point contact (Figure 1c). In contrast to the national method, the conditions were partially modified according to the actual situation. Every test was performed at least three times with a rotating rate of 1200 rpm/min, a load of 196 N, and a temperature of 50 ℃ under 60 min test duration. There are four GCr15 steel balls with a diameter of 12.7 mm (HRC 59–61) in total, the lower three balls remain stationary and the upper one is fixed by a clamp. After the end of the test, the wear scar diameter (WSD) was measured by using a digital-reading optical microscope with an accuracy of ±0.001 mm, and the average WSD was calculated by computer.

In addition, the tribological properties of Ni-doped lithium grease were evaluated under the ball-on-disk reciprocating model at 50 ℃ on the TE77 reciprocating friction tester (Figure 1c). The corresponding contact pressure was 40 N, the frequency was 3 Hz and the stroke length was 10 mm. The upper specimen was a GCr15 steel ball with a diameter of 10 mm, and the lower GCr15 steel disk with specifications of 58 mm × 38 mm × 4 mm. Each test was repeated at least three times to ensure the accuracy of the data. All the balls and disks were washed with PE and alcohol by ultrasonic vibration cleaners before the tests.

### 2.4. Characterization

#### 2.4.1. Characterization of Nanometer Ni and Grease

The morphology and agglomeration state of nanometer Ni were investigated by a transmission electron microscope (TEM, JEOL JEM200, Tokyo, Japan) and a scanning electron micrograph (SEM, HITACHI, SU8100, Tokyo, Japan). The size of nanometer Ni was measured by nano measurement software. The structure characteristics of nanometer Ni were evaluated using X-ray diffraction (XRD, Rigaku, Smart Lab, Tokyo, Jpan) analysis. A KRUSS DSA30R interfacial rheometer was applied to measure the contact angle of the nanometer Ni on the metal surface. The above process involved submerging the steel substrate in a lubricant with or without 0.2 wt% nanometer Ni for 30 min at 50 °C. The plate was rinsed with toluene and dried in the air. Then the equal amount of PAO4 was dropped on the plate. The contact angle between the base oil and the metal plate was observed and recorded automatically [22]. Meanwhile, the change of contact angle with time was recorded.

SEM was also used to investigate the thickener structure of the grease sample with spray gold treatment. Before the test, we should remove the base oil in the grease by multiple ultrasonic treatments and centrifugation for the mixture of grease and PE, and obtain the thickener part. The dispersion degree of nanometer Ni in grease was observed by a polarizing microscope.

#### 2.4.2. Characterization of Worn Surface

To investigate the morphology of the worn surfaces and the wear degree of the tribological test, a Contour GT-K white-light interferometer (Bruker, Billerica, MA, USA) and SEM were used. Before the tests, the steel balls and plates were cleaned by ultrasonic vibration with PE for 10 min and blown dry with N_2_ gas. Furthermore, to interpret the lubricating mechanism, the binding energies of typical elements on the worn surface were tested on an energy dispersive spectroscopy (EDS, Aztec X-MaxN 80, Oxford Instruments, Abingdon, UK) and an X-ray photoelectron spectrometer (XPS, AXIS Ultra DLD multifunctional, Kratos Analytical, Manchester, UK) while Mg-Kα radiation was used as the excitation source with the reference of C1s at 284.6 eV as an internal standard.

## 3. Results and discussion

### 3.1. Characterization Results of Nanometer Ni

**Figure 3:**
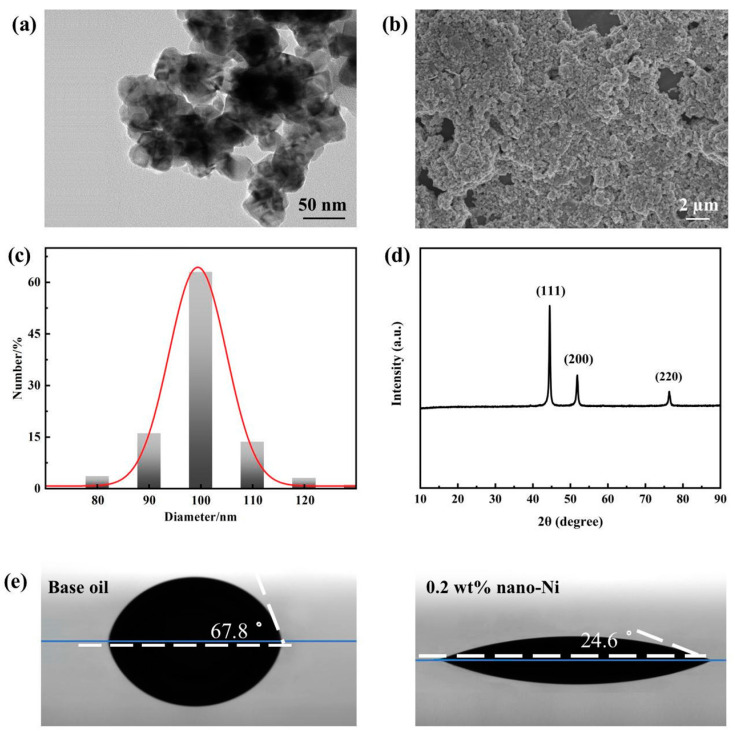
Characterization results of nanometer Ni: (**a**) TEM image; (**b**) SEM image; (**c**) particle size distribution; (**d**) XRD pattern; (**e**) contact angle diagram.

TEM and SEM images can reflect the morphology and dispersibility of the nanometer Ni. Figure 3a shows that the nanometer Ni has an obvious spherical morphology and the size was uniform. Meanwhile, the degree of agglomeration is also acceptable (Figure 3b). As shown in Figure 3c, the size distribution of nanometer Ni was in the range between 80 and 120 nm, while the average volume of particle size was around 100 nm. The XRD pattern of nanometer Ni is shown in Figure 3d. The (111), (220), and (220) crystal planes of Ni can be found at the obvious peaks at 44.5°, 51.8°, and 76.4°. Compared with the standard card (JCPDS Card No. 04-0850), the face-centered cubic (FCC) structure can be confirmed in the synthesized nanometer Ni, which can indicate the high purity of synthesized nanometer Ni without by-products.

The ability to adsorb onto a metal surface is essential to the effectiveness of additives. The contact angle measurements were performed to evaluate the adsorption behavior of nanometer Ni on the metal surface [22]. As shown in Figure 3e, after immersion in the base oil containing nanometer Ni, the contact angle between the steel plate and base oil decreased significantly, indicating that effective adsorption of the nanometer Ni on the metal surface can promote the rapid diffusion of oil on the metal surface and the formation of adsorption film. 

### 3.2. Characterization Results of Grease Sample

**Figure 4:**
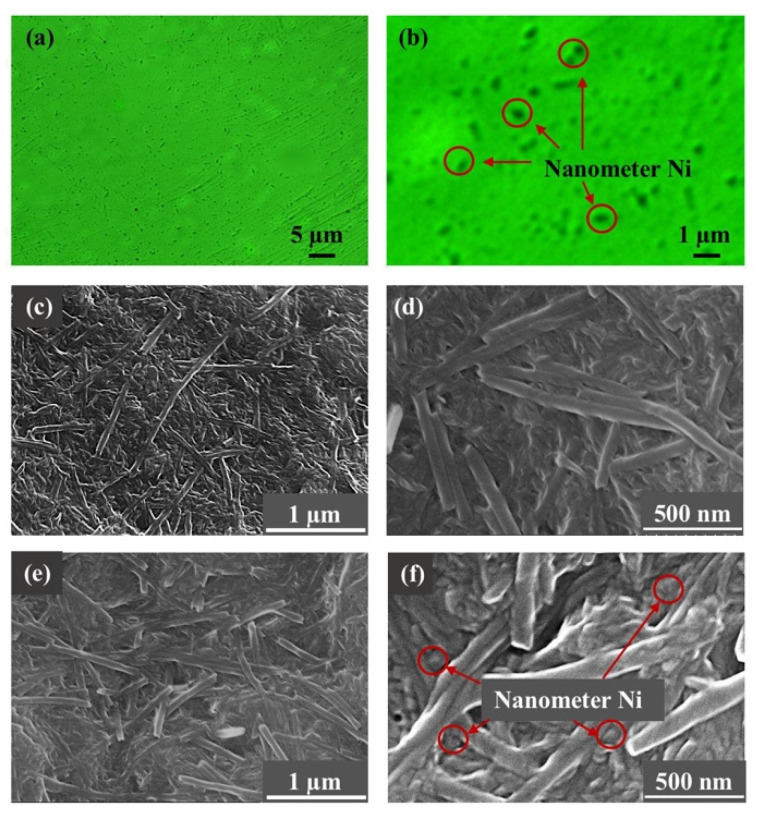
Characterization results of grease sample: (**a**,**b**) polarizing optical micrograph of 0.2 wt% Ni-doped lithium grease; SEM images of (**c**,**d**) pure lithium grease and (**e**,**f**) 0.2 wt% Ni-doped lithium grease.

The uniformity of dispersion directly determines the tribology performance of the doped grease. Since the grease is transparent under the polarizing microscope, the distribution of nanometer Ni (opaque black area) in the polarizing microscope image can be used as an index to evaluate the dispersion uniformity of the doped grease. As shown in Figure 4a,b, the black particles are evenly distributed in the overall image without large-scale agglomeration, which indicates that the composition of prepared Ni-doped lithium is uniform and can be further studied. The SEM images in Figure 4c-d show a tight thickener network of pure lithium grease. The fiber bundles constituting the thickener network are very long and show strip and spiral morphology. A tight network structure invested lithium grease with a strong constraint capability of base oil. The addition of nanometer Ni leads to a decrease in the fiber length (Figure 4e,f), which is speculated to be caused by the shearing action. Although all grease samples have gone through the same grinding operation, the contact between the nanoparticles leads to the formation of many small shear surfaces inside the Ni-doped grease. Therefore, the thickener has been more strongly sheared during the grinding process than in the pure grease, resulting in a reduction of the fiber bundle length. At the same time, it can be observed that the nanoparticles were distributed among the fiber bundles.

The physical properties are very important parameters for grease. The drop point is defined as the temperature when the grease changes from semi-solid to liquid, which is an indicator of the high-temperature performance of the grease. Cone penetration is an index to measure the consistency and hardness of grease. Pressure oil separation is used to reflect the colloidal stability of grease at 25 °C. Table 2 exhibits the results of the physical tests conducted on the pure grease and 0.2 wt% Ni-doped greases. There was no significant difference between pure lithium grease and Ni-doped lithium grease on the drop point and cone penetration tests. It is speculated that this consequence comes from the low Ni concentration in doped greases, which is inadequate to form a continuous structure in the greases. Interestingly, the rate of oil separation shows an obvious divergence. Under the same pressure, pure lithium grease has barely any oil separation, while Ni-doped grease has a small amount of oil separation. This is consistent with the structure of grease (Figure 3c,d). The shear action between nanoparticles can destroy the tight thickener network to a certain extent, reducing the length of the thickener fiber bundle, and then diminishing the binding ability of grease to base oil. As a result, a certain amount of base oil will exude from Ni-doped grease under the action of external force. At the same time, the base oil is essential to the formation of a continuous oil film, thus an appropriate oil separation is crucial to the tribological performance of the grease. Otherwise, the friction failure will be caused by a lack of oil.

### 3.3. Tribological Tests Results on Four-Ball Friction Tester

**Figure 5:**
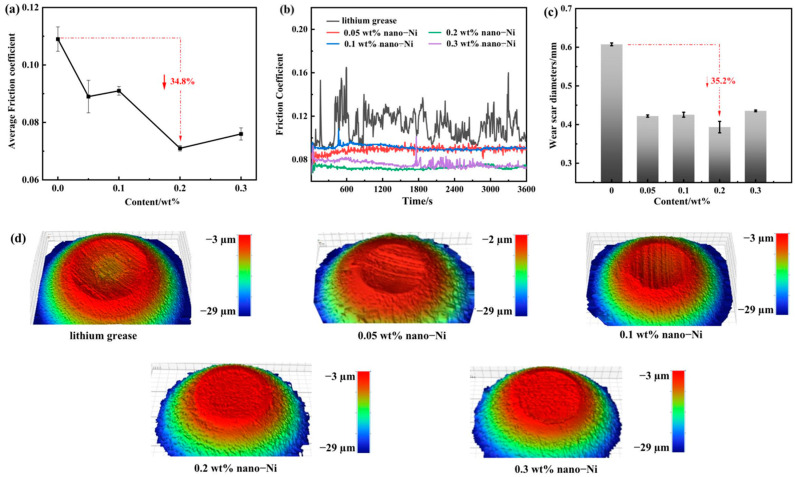
Tribological properties of the pure lithium grease and Ni-doped lithium grease under point-to-point contact: (**a**) Average COF; (**b**) COF curves; (**c**) Average WSD; (**d**) WLI morphologies of wear scar.

For the tribological performance under point-to-point contact of lithium grease, the concentration of nanometer Ni shows a significant effect. Figure 5a,b depicts the friction coefficient (COF) of the grease sample. Pure lithium grease shows a large COF and unstable friction state. In the range of 0.05–0.2 wt%, the increase of the nanometer Ni concentration can promote the decrease of the COF, and then increase inconspicuously with the concentration at 0.3 wt%. When the concentration is 0.2 wt%, Ni-doped grease reaches the minimum COF, which is 34.8% lower than that of pure grease, and the friction process is very stable without mutation. The results show that nanometer Ni can improve the friction-reducing performance of the grease as an additive under the point-to-point contact mode. Figure 5c,d shows the worn condition of the grease sample. With the increase of the concentration, the changing trend of WSD of steel ball surface is the same as that of COF (Figure 5a,b). Compared with pure lithium grease, the anti-wear ability of 0.2 wt% Ni-doped lithium grease is increased by 35.2%. For pure lithium grease, the obvious wear (localized fracture), plastic deformation, and random wide grooves can be discovered on the surface. In comparison, the wear spot of the 0.2 wt% Ni-doped lithium grease was the roundest with the smallest diameter, while a smooth and shallow scar with narrow and slight furrows was observed in the high-resolution image.

### 3.4. Tribological Test Results under Point-to-Flat Contact

**Figure 6:**
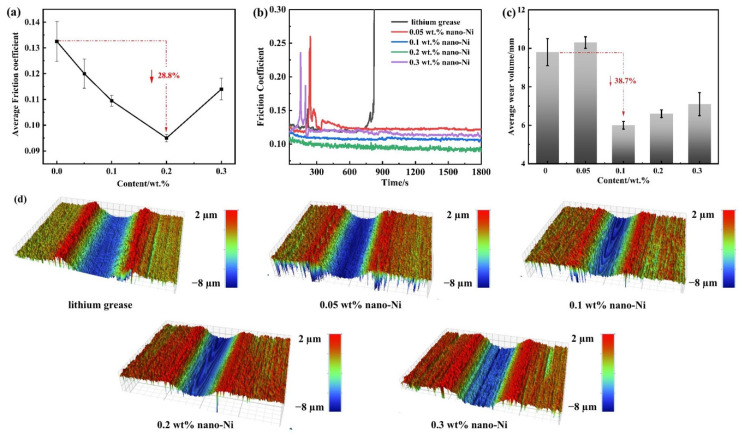
Tribological properties of the pure lithium grease and Ni-doped lithium greases under point-to-flat contact: (**a**) Average COF; (**b**) COF curves; (**c**) Average wear volume; (**d**) WLI morphologies of wear surface.

To further explore the effect of nanometer Ni on the tribological properties in lithium grease under point-to-flat contact, a TE77 reciprocating friction tester was used to measure the properties of Ni-doped grease (Figure 6). The variation trend of COF with the Ni content is similar to point-to-point contact. The friction failure of pure lithium grease occurred around the 800th second, which was caused by the rupture of the oil film. The minimum COF of Ni-doped grease appears at 0.2 wt% content, the friction-reducing capacity is 28.8% higher than pure lithium grease, while the COF curve decreases steadily during the experiment. The increase of COF at 0.3 wt% can be considered as the inordinate aggregation of nanometer Ni on the contact area. The anti-wear performance of the grease can be evaluated by the observation of the wear surface morphology after the friction test (Figure 6c,d). The depth and width information of the wear mark can be provided intuitively by the three-dimensional image. The friction leads to obvious wide trenches and severe destruction on the wear area by pure grease lubrication, while the wear mark of 0.05 wt% Ni-doped lithium grease is deep and smooth. When the concentration of nanometer Ni increases to 0.1wt%, the volume of the worn mark reduces significantly. The continuous increase in content resulted in narrow and deep wear marks. Therefore, 0.1 wt% Ni-doped lithium grease has the best anti-wear ability under point-to-flat contact. In conclusion, the tribology performance of Ni-doped grease is particularly significant compared with lithium grease under point-to-flat contact, which shows that the strength of oil film can be improved with nanometer Ni.

According to these data, we can infer that the concentration of nanometer Ni can optimize the tribology performance of lithium grease. Under point-to-point and point-to-flat contact, the optimum concentration of Ni-doped lithium grease is 0.2 wt%. 

### 3.5. SEM Analysis

**Figure 7:**
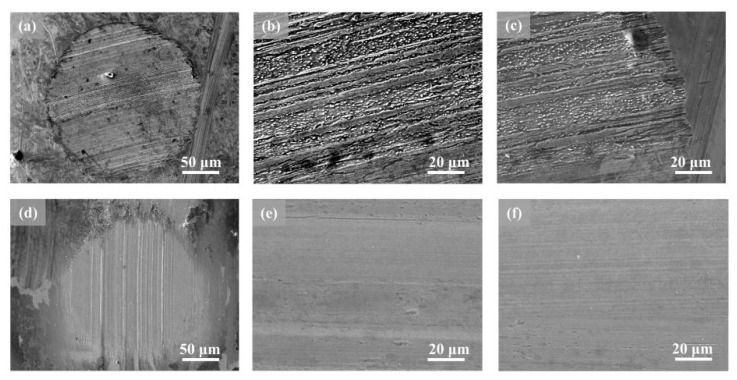
SEM morphologies of wear scars lubricated by (**a**–**c**) pure lithium grease and (**d**–**f**) 0.2 wt% Ni-doped lithium grease after four-ball tribology tests.

From the SEM morphologies of the worn surfaces (Figure 7), the obvious furrows, scratches, and pits can be detected on the surface which was lubricated by the pure lithium grease. When the nanometer Ni (0.2 wt%) was added, the number of groove plows on the surface of the friction pair after friction is significantly reduced, while the surface becomes flat. The flagrant contrast between the two samples exhibits that the addition of nanometer Ni can improve the anti-wear performance of lithium grease significantly. It is speculated that this mainly originates from the filling effect of nanometer Ni, which can be absorbed in the pits of the friction pair to form a solid physical layer with low shear stress in the pits of the friction pair [23,24]. Meanwhile, the spherical structure of nanometer Ni can convert the sliding friction to rolling friction, thus reducing the friction coefficient and improving the bearing capacity [25,26]. Furthermore, the rough surface was mechanically polished with nanometer Ni. Thereby the smoothness and flatness were increased, improving the tribology properties of the grease [27].

### 3.6. EDS Analysis on the Worn Surface

**Figure 8:**
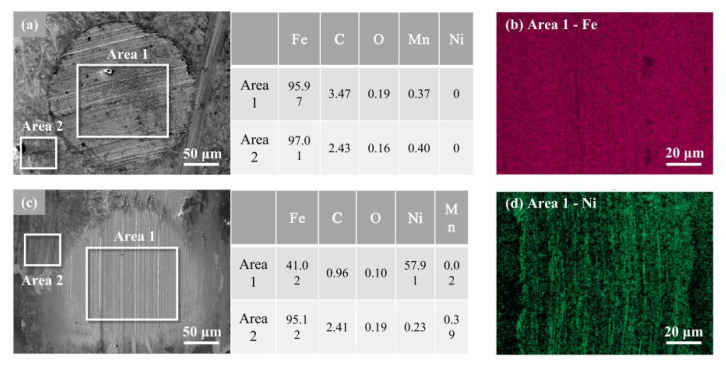
EDS mapping images of the worn mark surfaces after four-ball test lubricated by (**a**,**b**) lithium grease; (**c**,**d**) 0.2 wt% Ni-doped lithium grease.

The distribution of elements was scrutinized in the EDS mapping image of the surface after four-ball friction (Figure 8), which was carried out on the worn area (area 1) and non-worn area (area 2). Figure 8a,b shows a small distinction in the element content between area 1 and area 2 with the pure lithium grease lubrication, which was mainly composed of the metal base element Fe. The high content of the C element due to the strong wear was also detected (area 1). Figure 8c,d shows the mapping image of the surface with the Ni-doped grease lubrication. The main distinction between the worn area (area 1) and non-worn area (area 2) derives from Fe and Ni elements. In the worn area, it is evident that the concave part of the wear mark is covered evenly with Ni element, which comes from the nanometer Ni in the doped grease. According to the element types found in worn surfaces, it could be inferred that the nanometer Ni can effectively penetrate the friction pairs from the lithium grease to form a lubricating film. The striking contrast between pure lithium and doped grease further verifies the existence of the deposited film [28], which is consistent with the SEM results (Figure 7d–f). Additionally, considering the steel ball surface has been cleaned with PE before EDS analysis, this shows that the deposited film of nanometer Ni is stabilized.

### 3.7. XPS Analysis on the Worn Surface

**Figure 9:**
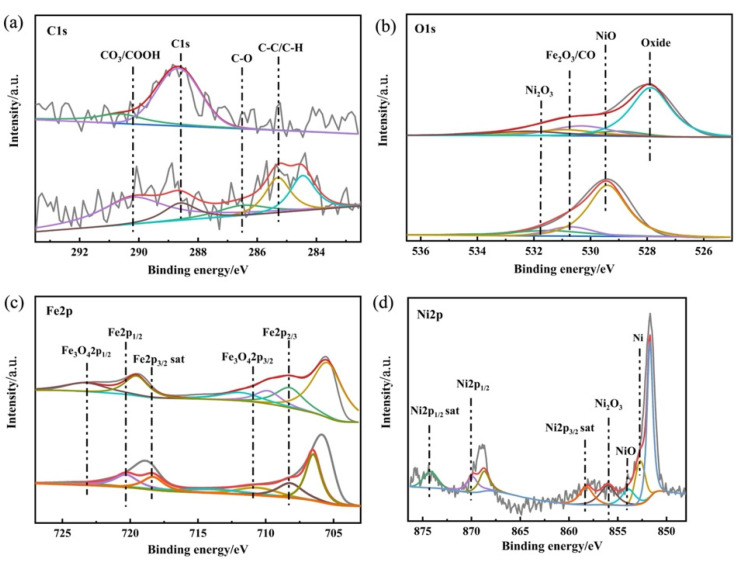
XPS spectra of (**a**) C1s, (**b**) O1s, (**c**) Fe2p, and (**d**) Ni2p on the worn scars of steel balls lubricated by pure lithium grease and 0.2 wt% Ni-doped lithium grease after four-ball tribology tests.

The XPS analysis was conducted to further clarify the chemical states of the typical elements on the worn surfaces and further investigate the lubrication mechanism of the nanometer Ni compound with lithium grease. Figure 9 shows the high-resolution XPS spectra of C1s, O1s, Fe2p, and Ni 2p of wear scar on the GCr15 steel ball lubricated with lithium grease and 0.2 wt% Ni-doped lithium grease. The peak at 288.3 eV is indicative of C in the carboxyl group. The peak at 285.2 eV is attributed to the C–C bonding. The peak at high energy of 529.6 eV is associated with oxygen in the NiO. The peak at 530.8 eV is indicative of the oxygen in the Fe_2_O_3_. The peak appearing around 531.7 eV is attributed to Ni_2_O_3_ [29]. The peak of Fe2p appears at a binding energy of 708.2 eV, which is identified as the FeO. The peak at 711.1 eV is indicative of the Fe2p in the Fe_2_O_3_. In the high-resolution XPS spectra of Ni2p on the steel ball, which was lubricated with 0.2 wt% nano-Ni nanoparticles, the peak appearing at 852.9 eV is attributed to the NiO, while the peak at 856.2 eV is associated with Ni_2_O_3_. Based on these data, we can confirm that complicated tribochemical reactions were involved during the friction process. The Ni-doped lithium grease can generate a surface protective film composed of NiO, Ni_2_O_3_, ferrites, and compounds containing the C-O bonding on the rubbing surface, which significantly contributed to the friction reduction and anti-wear properties [30].

### 3.8. Mechanism of Lubrication

**Figure 10:**
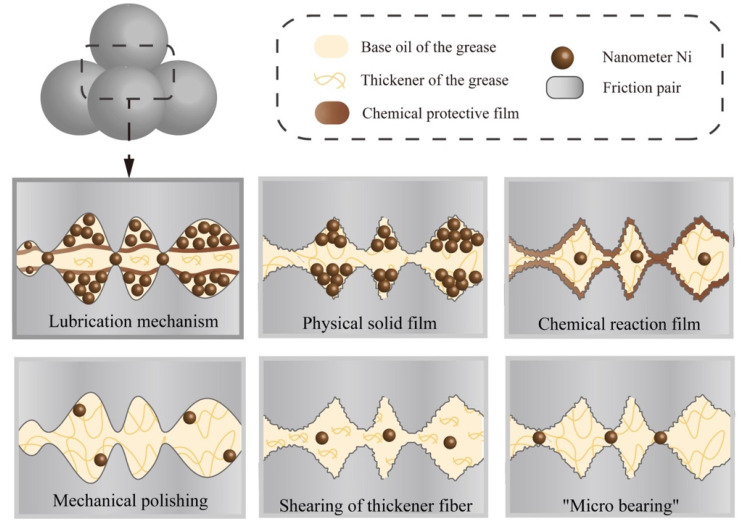
Schematic of the lubrication mechanism of nanometer Ni as an additive in lithium grease.

Based on the examination and characterization results, we now speculate on the possible lubrication mechanism of the nanometer Ni, which is constituted of five functions (Figure 10). The first is generating a physically solid film. Due to the high surface energy, nanometer Ni adsorbed on the rubbing surface and formed the physical adsorption films [31,32,33]. Meanwhile, the diffusion of the nanometer Ni formed a diffusion layer and a penetration layer with friction-reducing ability on the metal surface (Figure 7 and Figure 8). The second is generating a chemical-reaction film. The high thermal energy induced by friction resulted in the high temperature of the surface, which may promote the chemical reaction of the nanometer Ni to a certain extent [34,35,36,37]. Thereby a reaction film with anti-wear and friction-reducing characteristics was formed on the friction pair (Figure 9). The third is mechanical polishing [38,39]. Under external pressure, nanometer Ni in the doped grease destroyed the partially nanoscale bulge of the rough surface and polished them mechanically. The smooth surface increases the contact area between the friction pairs and prevents the occurrence of serious abrasive wear. Thereby the COF was reduced (Figure 7). The fourth is shearing the thickener fiber. The shearing behavior of nanometer Ni damaged the fiber structure of the grease thickener to a certain extent, resulting in a relaxed network and decreased fiber length, thus the binding capacity of the thickener to the base oil was reduced. The base oil maintains the continuity of the oil film (Table 2) and avoids lubrication failure (the COF curve of pure lithium grease in Figure 6). Therefore, nanometer Ni improved the capacity of oil separation and played a stable lubrication role (the COF curve of Ni-doped lithium grease in Figure 5 and Figure 6). The fifth is "micro bearing". The small and incomplete crystal nucleus of nanoparticles brought about the dislocation and distortion in the grain structure. Under a high load, the slipping of the lattice was easily induced, hence the spherical structure of nanometer Ni can convert the sliding friction to rolling friction, thus improving the lubrication property, which is similar to "micro bearings" on the contact surface. The lubrication ability of nanometer Ni in lithium grease is attributed to the above five possible functions, which broadens working conditions and prolongs service life in comparison with pure grease [40,41].

## 4. Conclusion

In summary, nanometer Ni with uniform particle size was synthesized by the direct reduction method, and Ni-doped grease was prepared by mechanical stirring, ultrasound vibration, and three-roll grinding. The friction tests under different working conditions were designed to investigate the tribological properties of nanometer Ni. The possible lubrication mechanism of nanometer Ni as a lubricating additive for lithium grease was discussed and analyzed in detail.

(1) Under both point-to-point and point-to-flat contact modes, the nanometer Ni exerts excellent lubrication effects as an additive in lithium grease, with the optimized performance achieved at the concentration of 0.2 wt%.
(2) It is speculated that the lubrication mechanism of the nanometer Ni in lithium grease is constituted of five functions: generating a physically solid film, generating a chemical-reaction film, mechanical polishing, and shearing the thickener fiber, and "micro bearing". The synergy of these functions significantly improves the friction-reducing and anti-wear properties of grease.

## Figures and Tables

**Table 1:** Test conditions used for physical properties of grease sample.

Physical Characterization	Standard	Duration (min)	Quantity of Grease Required (g)	Temperature (°C)	Heat Rate (°C/min)
Cone penetration	GB/T 269—91	0.0833	40	25	-
Drop point	GB/T 4929	Not specified	2	0 to +300	5–8
Oil separation rate	GB/ T392	30	Not specified	25	-

**Table 2:** The physical characterization results.

Tests	Cone Penetration (1/10 mm)	Drop Point (°C)	Oil Separation Rate (%)
Pure lithium grease	365.6	168.3	Barely
0.2 wt% Ni-doped lithium grease	366.5	169.1	1.7

## Data Availability

Not applicable.
