# Correction: Wang et al. Tribological Properties and Lubrication Mechanism of Nickel Nanoparticles as an Additive in Lithium Grease. *Nanomaterials* 2022, *12*, 2287

**DOI:** 10.3390/nano13202792

**Published:** 2023-10-19

**Authors:** Jiabei Wang, Hong Zhang, Wenjing Hu, Jiusheng Li

**Affiliations:** 1 Laboratory for Advanced Lubricating Materials, Shanghai Advanced Research Institute, Chinese Academy of Sciences, Shanghai 201210, China; wangjiabei2019@sari.ac.cn (J.W.); xyyyswlp@163.com (H.Z.); 2 University of Chinese Academy of Sciences, Beijing 100049, China

## Error in Figures

In the original publication [1], there was a mistake in Figure 3 as published. We inadvertently selected an incorrect TEM image for Figure 3a, which was captured from the sample of modified nanometer Ni instead of the nanometer Ni. We replace it with the correct TEM image representing the pristine nanometer Ni particles to this correspondence. The corrected Figure 3 appears below.

**Figure 3:**
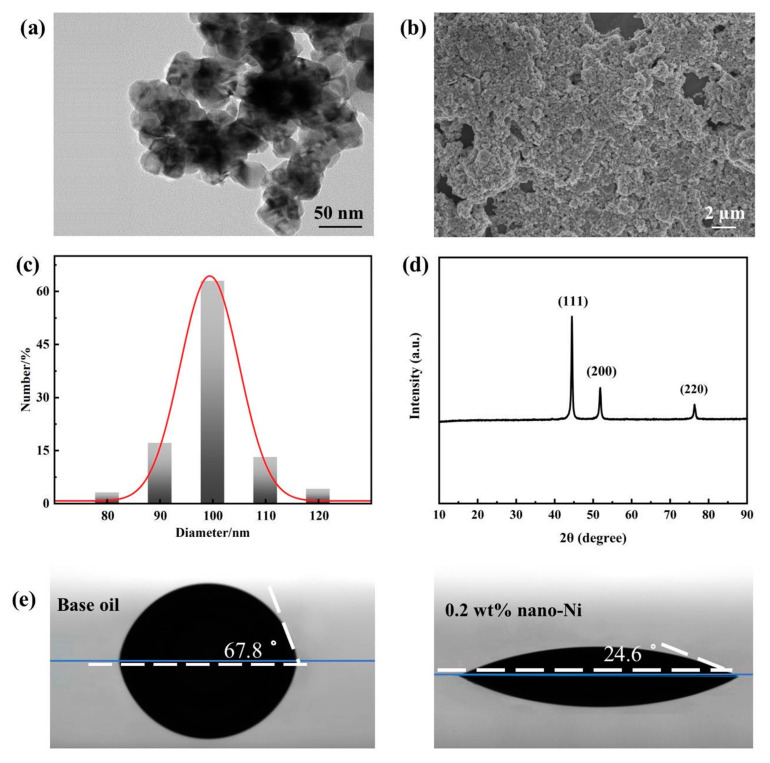
Characterization results of nanometer Ni: (**a**) TEM image; (**b**) SEM image; (**c**) particle size distribution; (**d**) XRD pattern; (**e**) contact angle diagram.

In the original publication, there was an insufficiency in Figure 5 as published. Compared to Figure 6d, the wear diagram in Figure 5d provides only two-dimensional information. The corrected Figure 5 appears below. 

**Figure 5:**
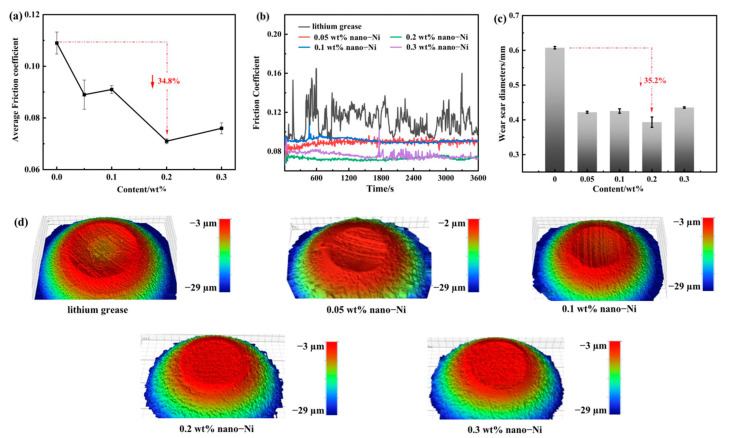
Tribological properties of the pure lithium grease and Ni-doped lithium grease under point-to-point contact: (**a**) Average COF; (**b**) COF curves; (**c**) Average WSD; (**d**) WLI morphologies of wear scar.

The authors state that the scientific conclusions are unaffected. This correction was approved by the Academic Editor. The original publication has been updated.
